# Searching for shelter in a ferruginous cave? A new species of *Pasipha* from a plateau in the Brazilian savanna (Platyhelminthes, Tricladida)

**DOI:** 10.3897/zookeys.776.26308

**Published:** 2018-07-26

**Authors:** Ana Leal-Zanchet, Alessandro Marques

**Affiliations:** 1 Instituto de Pesquisas de Planárias and Programa de Pós-Graduação em Biologia, Universidade do Vale do Rio dos Sinos, 93022-750 São Leopoldo, Rio Grande do Sul, Brazil

**Keywords:** Geoplaninae, land planarians, Neotropical region, taxonomy

## Abstract

In a fauna survey in the eastern margin of Serra do Espinhaço Plateau, in an area belonging to the Brazilian savanna (Cerrado phytophysiognomy), a land flatworm was sampled in a ferruginous cave. Anatomical and histological analyses indicated that it belongs to a new species of the genus *Pasipha*, which is herein described. The new species shows an almost homogenous dark brown dorsal pigmentation, eyes spreading over the dorsal surface, a collar-shaped pharynx, and a prostatic vesicle with two portions separated by a canal. It differs from similar species mainly by anatomical and histological details of the ejaculatory duct, as well as male and female atria. The flatworm shows no troglomorphic traits and was collected once in the entrance zone of the cave. Hence, despite representing the first land flatworm species described from a Neotropical cave, we consider that its occurrence in the cave is probably occasional, using it as a shelter.

## Introduction

The genus *Pasipha* Ogren & Kawakatsu, 1990 encompasses 25 species, most of them known from southeast and southern Brazil (Carbayo et al. 2013; Leal-Zanchet et al. 2012; Amaral and Leal-Zanchet 2016; Negrete and Brusa 2016, 2017; Amaral et al. 2018). Most species, including the type-species, *Pasipha pasipha* (Marcus, 1951), were described from areas of dense ombrophilous forest of the states of São Paulo, Rio de Janeiro and Minas Gerais, in southeast Brazil (Riester 1938; Marcus 1951; E.M. Froehlich 1955). Other 10 species occur in areas of mixed ombrophilous forest and semi-deciduous or deciduous forests from southern Brazil and Argentina (Froehlich 1959; Leal-Zanchet et al. 2012; Amaral and Leal-Zanchet 2016; Negrete and Brusa 2016; Amaral et al. 2018), one of them also occurring in the Amazonian biome (Froehlich and Froehlich 1972).

In a recent fauna survey in the eastern margin of Serra do Espinhaço Plateau, belonging to the Brazilian savanna (Cerrado phytophysiognomy), southeastern Brazil, a flatworm with elongate body and parallel margins was collected in a ferruginous cave. This specimen was assigned to the genus *Pasipha* and is herein described as a new species.

## Materials and methods

**Figure 1.**
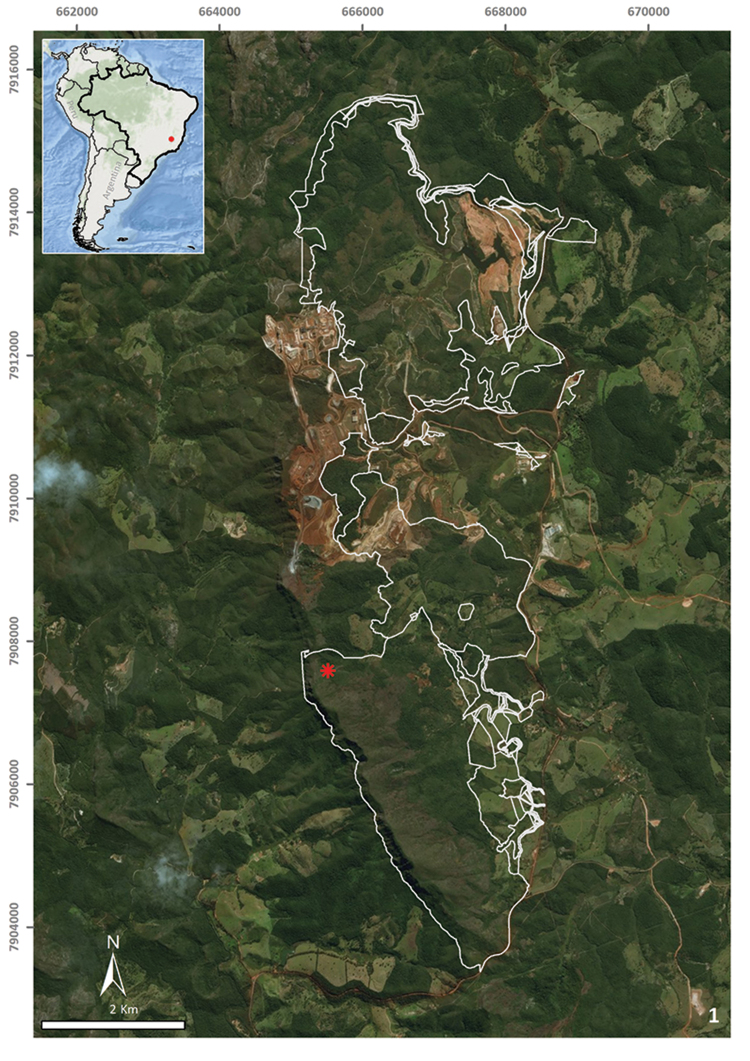
Location of the type locality of *Pasipha ferrariaphila* sp. n., represented by a ferruginous cave, Conceição do Mato Dentro, state of Minas Gerais, Brazil. The asterisk indicates the cave location; the outline indicates areas impacted by mining exploitation projects.

A single specimen was collected during the day by direct sampling in the entrance zone of a ferruginous cave (CSS-0004) in Conceição do Mato Dentro (18°55'02.2"S, 43°25'42.4"W), at an altitude of 931 m a.s.l., in the state of Minas Gerais, southeastern Brazil (Fig. 1). The land flatworm was fixed in 70% ethyl alcohol during field work. The preserved specimen was analysed regarding colour pattern, body shape, and dimensions and then photographed under a stereomicroscope. Methods described by Rossi et al. (2015) were used for histological processing of the material and analysis of external and internal characters. The material was sectioned at intervals of 6 µm and stained with Goldner’s Masson or Haematoxylin and Eosin.

Type-material is deposited in the Helminthological Collection of Museu de Zoologia da Universidade de São Paulo, São Paulo, São Paulo State, Brazil (**MZUSP**).

Abbreviations used in the figures:

**cmc** common muscle coat

**cov** common glandular ovovitelline duct

**cs** creeping sole

**de** dorsal epidermis

**di** dorsal insertion of pharynx

**dm** dorsal cutaneous musculature

**dpv** distal portion of prostatic vesicle

**e** eyes

**ed** ejaculatory duct

**eg** erythrophil secretion

**fa** female atrium

**fc** female canal

**go** gonoduct

**h** parasitic helminths

**i** intestine

**lu** pharyngeal lumen

**ma** male atrium

**mo** mouth

**ms** median stripe

**n** nerve plate

**om** outer musculature of pharynx

**ov** ovovitelline duct

**pp** pharyngeal pouch

**ppv** proximal portion of prostatic vesicle

**pv** prostatic vesicle

**r** rhabdites

**sbm** sub-intestinal transversal muscles

**sd** sperm duct

**sg** shell glands

**sm** sensory margin

**spm** supra intestinal transversal muscles

**t** testes

**v** vitellaria

**vi** ventral insertion of pharynx

**vm** ventral cutaneous musculature

## Taxonomic description

### Family Geoplanidae Stimpson, 1857

#### Subfamily Geoplaninae Stimpson, 1857

##### *Pasipha* Ogren & Kawakatsu, 1990

###### Pasipha ferrariaphila sp. n.

Taxon classificationAnimaliaTricladidaGeoplanidae

http://zoobank.org/FA4C4AA2-0064-40AA-848D-C76C7ADFC828

####### Type material.

Holotype MZUSP PL 2141: leg. *Carste Ciência e Ambiente*, 16 January 2014, Conceição do Mato Dentro (18°55'02.2"S, 43°25'42.4"W; altitude 931 m a.s.l.), state of Minas Gerais (MG), Brazil – anterior tip: transverse sections on 8 slides; anterior region at the level of the ovaries: sagittal sections on 7 slides; pre-pharyngeal region in two fragments: transverse sections on 4 slides and sagittal sections on 6 slides; pharynx: sagittal sections on 5 slides; copulatory apparatus: sagittal sections on 8 slides.

####### Type-locality.

Conceição do Mato Dentro, state of Minas Gerais (MG), Brasil.

####### Diagnosis.

*Pasipha ferrariaphila* is characterised by almost homogeneous dorsal pigmentation pattern, eyes spreading over the dorsal surface, collar-shaped pharynx, prostatic vesicle with two portions separated by a canal, ejaculatory duct long and spacious, male and female atria separated by a constriction and female atrium spacious, long and with a strongly developed circular musculature in its proximal part, resembling a sphincter.

####### Description.

**Figures 2–3.**
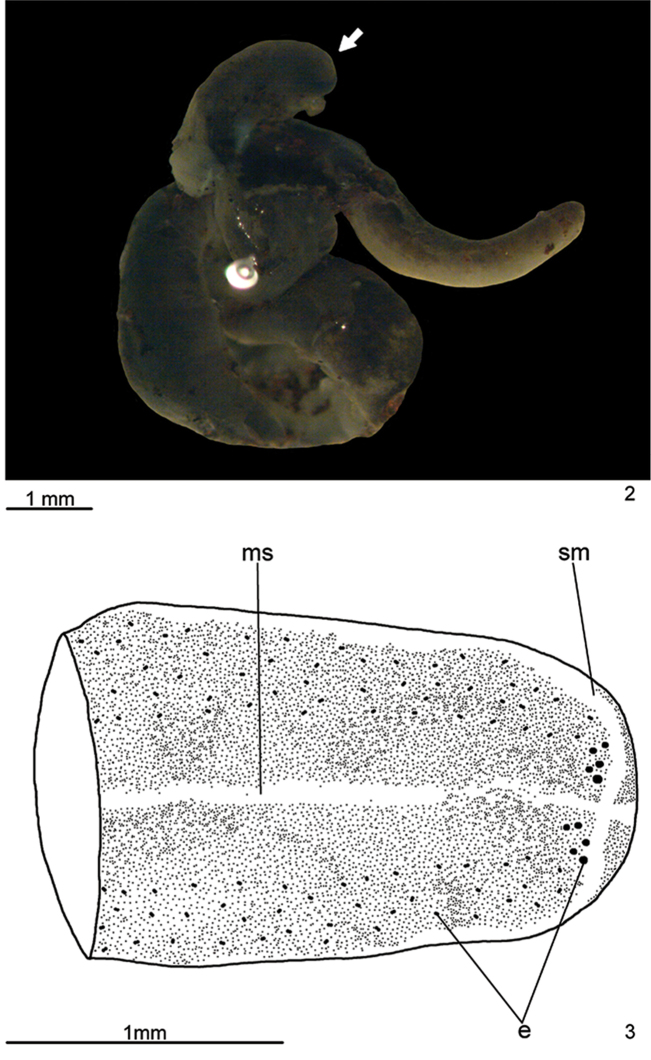
*Pasipha ferrariaphila* sp. n., holotype, dorsal view, **2** general view of preserved specimen **3** schematic drawing of the anterior region of body. The arrow indicates the anterior extremity. Eyes were drawn based on observations carried out under both stereomicroscope and microscope.

Body elongate with parallel margins; anterior tip rounded and posterior tip pointed (Figs 2–3). After fixation, length of 22 mm, maximal width of 2 mm, and maximal height 0.7 mm. Mouth at 77% of body length and gonopore at 82% of body length.

Fixed specimen with dorsal surface covered by fine, almost homogeneous dark brown pigmentation over light brown ground colour, which is discernible under stereomicroscope on a thin, almost imperceptible median stripe (Fig. 3). Ventral surface pale yellow.

Eyes, initially monolobate (pigment cups of 15–25 µm) and disposed in an irregular row, surround anterior tip (Fig. 3). After that, some eyes become bilobed (pigment cups of 10–15 µm) and spread onto dorsal surface of body. Towards posterior end, eyes gradually becoming sparser.

**Figures 4–8.**
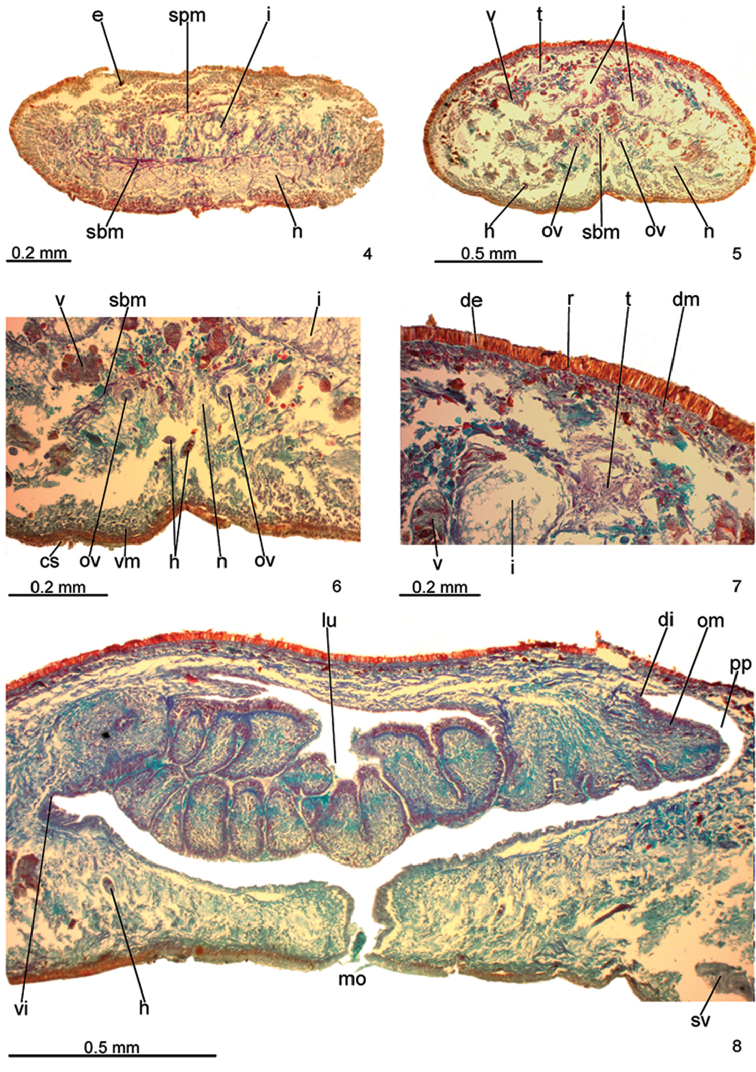
*Pasipha ferrariaphila* sp. n., holotype. **4** anterior region, transverse section **5–7** pre-pharyngeal region, transverse sections **8** pharynx, sagittal section.

Sensory pits, as simple invaginations (20–40 μm deep), contour anterior tip and occur ventro-marginally in a single row (Fig. 4) in approximately the anterior 1/6 of body. Creeping sole occupies almost whole body width.

Four gland types discharge through dorsal epidermis and body margins of pre-pharyngeal region: abundant rhabditogen cells with xanthophil secretion (Figs 5–7), numerous erythrophil glands with coarse granular secretion of two types (with ovoid or rounded granules), and sparse cyanophil glands with amorphous secretion. Creeping sole receives three types of glands: cyanophil glands with amorphous secretion, rhabditogen cells with small rhabdites, as well as scarcer glands with rounded erythrophil granules. Glandular margin absent (Fig. 5). Glands discharging through anterior tip of body similar to those of pre-pharyngeal region.

Cutaneous musculature with usual three layers (circular, oblique, and longitudinal layers), longitudinal layer with small bundles (Figs 6–7). Mc:h 10%. Thickness of cutaneous musculature similar to that of epidermis. Ventral musculature (about 30 μm) two times thicker than dorsal musculature (about 15 μm) at sagittal plane in pre-pharyngeal region. Thickness of cutaneous musculature gradually diminishes towards anterior tip.

Mesenchymal musculature (Figs 6–7) poorly developed, mainly composed of three layers: (1) dorsal subcutaneous with oblique decussate fibres (about 2 fibres thick); (2) supra-intestinal transverse (about 2 fibres thick); (3) sub-intestinal transverse (about 3–4 fibres thick). Mesenchymal musculature thicker in cephalic region than in pre-pharyngeal region, especially sub-intestinal transverse layer (Fig. 4); thickness gradually diminishes towards anterior tip.

Pharynx collar-shaped, nearly 8% of body length, occupies almost all length of pharyngeal pouch. Pharyngeal dorsal insertion posteriorly shifted next to end of pharyngeal pouch. Mouth in median third of pharyngeal pouch (Fig. 8). Oesophagus absent.

**Figure 9.**
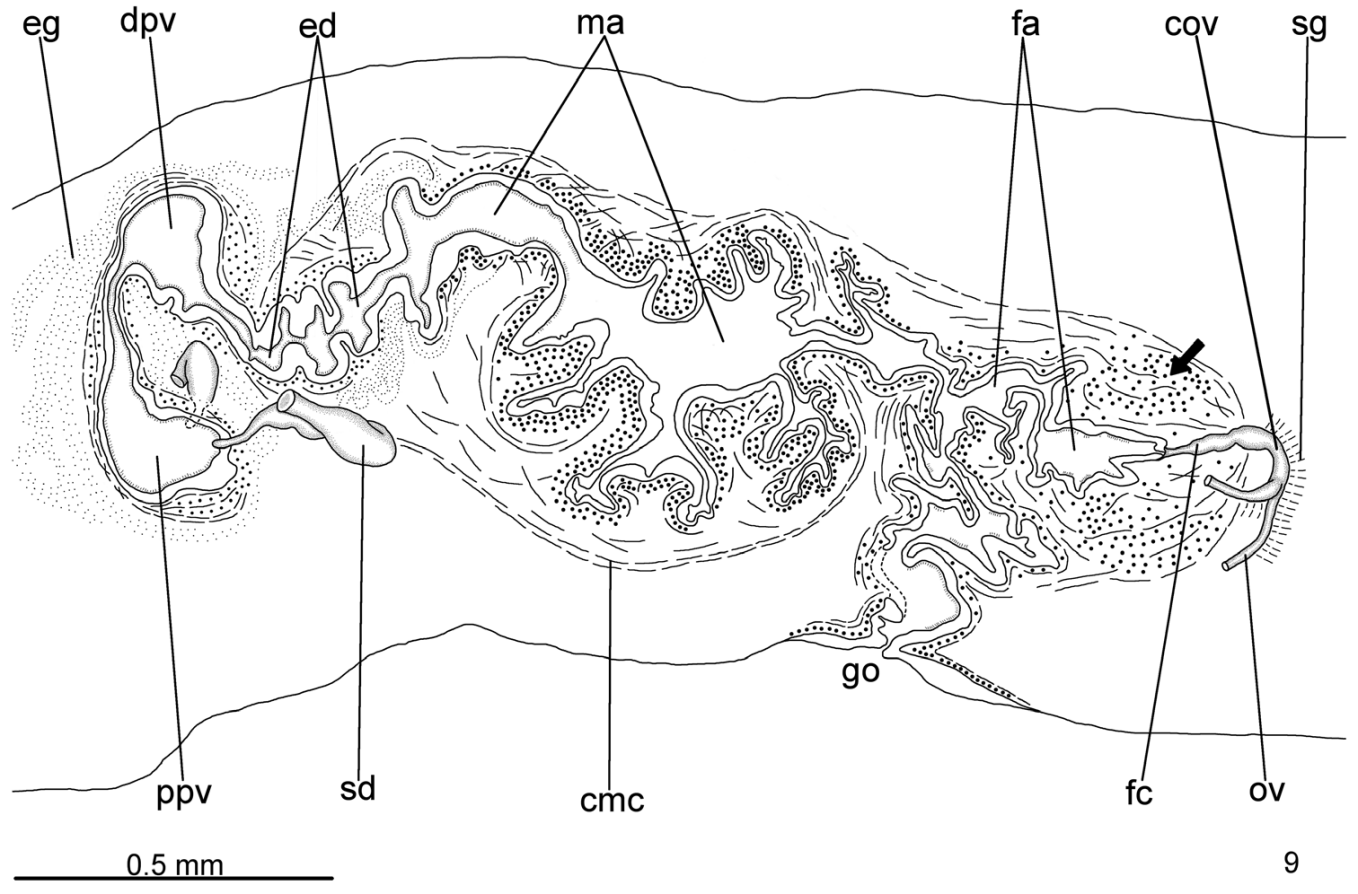
*Pasipha ferrariaphila* sp. n., holotype, sagittal composite reconstruction of copulatory apparatus. The arrow indicates the strong musculature in female atrium. Anterior to the left.

**Figures 10–14.**
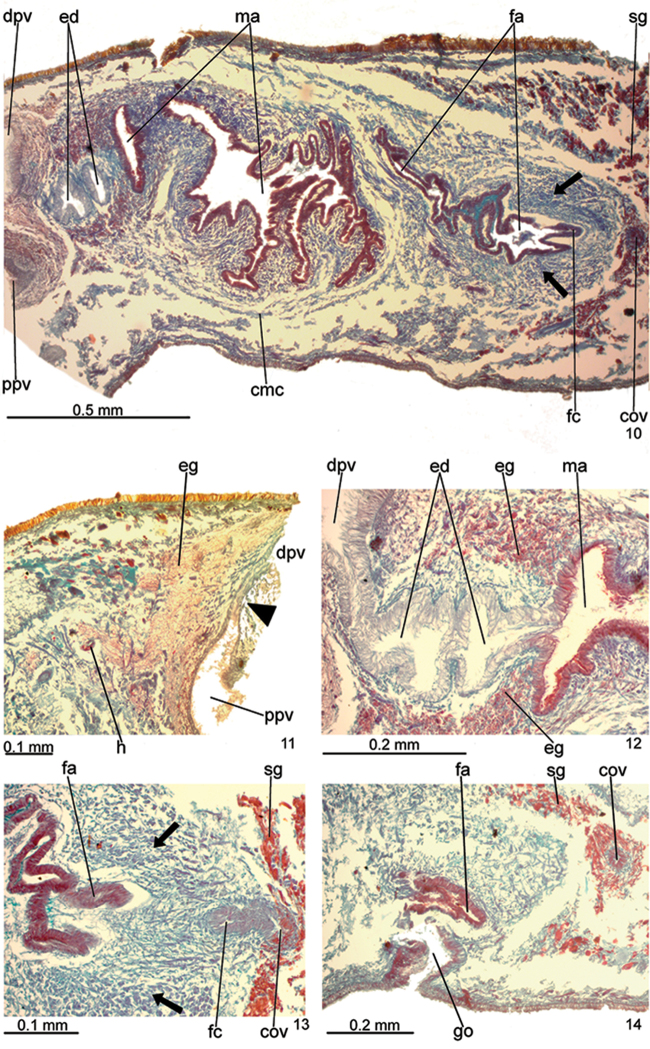
*Pasipha ferrariaphila* sp. n., holotype, copulatory apparatus in sagittal sections. **10** general view **11** proximal region of prostatic vesicle **12** ejaculatory duct and proximal portion of male atrium **13** proximal portion of female atrium **14** gonoduct. Arrows indicate the strong musculature in female atrium; the arrow head indicates the canal separating proximal and distal regions of prostatic vesicle. Anterior to the left.

Testes in two irregular rows on either side of body, located close to dorsal cutaneous musculature (Fig. 7). Testes begin at the same transversal level as ovaries, about 3.5 mm from anterior tip (16% of body length), and extend to near root of pharynx. Sperm ducts dorsal to ovovitelline ducts, laterally displaced, forming spermiducal vesicles laterally to pharynx. Behind pharynx, spermiducal vesicles well developed and sinuous, extending laterally to penis bulb. These vesicles recurve, ascend, and, subsequently, open through lateral walls of proximal portion of prostatic vesicle (Fig. 9). Large prostatic vesicle extrabulbar and not forked, close to pharyngeal pouch. This vesicle shows two portions united by a narrow canal: proximal portion oval-elongate, with a spacious lumen; distal portion globose with a narrower lumen (Figs 9–11). Ejaculatory duct sinuous, with irregular contour and ample lumen, arising from posterior region of prostatic vesicle and thereafter ascending to open into proximal portion of male atrium. Male atrium long with folded walls (Figs 9–10). Proximal region of male atrium, about anterior 1/4 of male atrium length, with narrower lumen (Figs 9–10; 12). Distal region of male atrium communicates with female atrium through a constriction (Figs 9–10).

Epithelial lining of prostatic vesicle ciliated and columnar, receiving coarse granular erythrophil or probably mixed secretion (erythrophil core and a chromophobic peripheral part), more abundant in its proximal portion. Distal portion of prostatic vesicle receives numerous amorphous, slightly cyanophil secretions. Muscularis of prostatic vesicle thick (20–35 µm thick), constituted of interwoven longitudinal, circular and some oblique fibres (Fig. 11). Canal uniting both portions of prostatic vesicle receives few coarse granular erythrophil secretions. Ejaculatory duct lined with ciliated, columnar epithelium, receiving openings from finely granular, cyanophil glands. Muscle coat of ejaculatory duct relatively thick (about 20 µm), mainly constituted of circular fibres. Male atrium lined with non-ciliated and erythrophil epithelium in distal region, ciliated and cyanophil in proximal region. Glands of distal region of two types: with coarse granular, erythrophil secretion and with amorphous, cyanophil secretion, whereas proximal region receives a third type with finely granular, erythrophil secretion. Muscularis of male atrium thick (50–60 µm), mainly comprised of circular fibres followed by some longitudinal fibres, diminishing in thickness and number of fibres in proximal region (20 µm).

Vitelline follicles, situated between intestinal branches, well-developed (Figs 5–7). Ovaries oval-elongate, measuring about 0.3 mm in length; they are situated dorsally to ventral nerve plate, about 3.5 mm from anterior tip (16% of body length). Ovovitelline ducts emerge dorsally from median third of ovaries and run posteriorly, close to median plane, immediately above ventral nerve plate. Distal sections of ovovitelline ducts run medially lateral to female atrium, with a slight asymmetry, the left ovovitelline duct contouring the atrial coat ventrally dislocated. They unite posteriorly to female atrium to form a C-shaped, ascending common glandular ovovitelline duct (Fig. 9). Female canal, horizontal, penetrates female muscle coat and opens into posterior-most part of female atrium. Female atrium ovoid with folded walls and narrow lumen (Figs 9–10; 13). Length of female atrium about 4/5 of male atrial length.

Female canal and atrium lined with columnar epithelium, sparsely ciliated in female canal. Glands of female atrium of two types: numerous glands with cyanophil, amorphous secretion and few glands with coarse granular erythrophil secretions. Female canal receives scant glands with coarse granular erythrophil secretion. Musculature of female atrium well developed, especially in proximal half (120 µm thick), composed mainly of circular fibres intermingled with some oblique fibres (Fig. 13).

Male and female atria with independent muscle coats (Figs 9–10), comprising longitudinal, oblique, and circular fibres. A constriction separates male and female atria (Figs 9–10). Gonoduct large and inclined backward at the sagittal plane (Figs 9; 14).

####### Ecology and distribution.

*Pasipha ferrariaphila* is known only from its type locality. It was sampled in an area situated in the eastern margin of Serra do Espinhaço Plateau, in southeastern Brazil. The area is covered by Brazilian savanna on rocky outcrops, also known as rupestrian complexes (Rapini et al. 2008, Oliveira et al. 2018), which occur associated with quartzite, sandstone, and itabirite above 900 m of altitude along the Serra do Espinhaço (Giulietti et al. 2000). The sampling site is the entrance zone of a ferruginous cave, representing 80% of the cave area. The sampling place is a low cavity (1.6 m high) with an area of 37 m^2^ and sandy soil covered by crushed ferruginous rocks. It is located in an area planned for mining activities, which is constituted by itabirite profoundly affected by such activities. Caves within iron formations are small and narrow, being formed by chemical, physical, and biological processes (Auler 2015). Ten samplings were conducted in the area between December 2010 and September 2014, but a single specimen of *P. ferrariaphila* was collected. Since this specimen shows no troglomorphic features and was collected only once in the entrance zone of the cave, we consider that its occurrence in the cave is probably occasional, using it as a temporary shelter.

####### Remarks.

The holotype, directly fixed in 70% ethanol during field work, showed a coiled body with some artefacts (Fig. 2), such as loss of the epidermis in some body parts, numerous lacunae in the mesenchyme and ovaries. In spite of that, the anatomy and most histological aspects were relatively well preserved. Nevertheless, the specimen may have some distortion in its anatomical features. The flatworm was parasitised by helminths (Figs 6; 8; 11).

####### Comparative discussion.

The new species herein described shows characteristics that match the diagnosis of the genus *Pasipha*, such as a body shape with parallel margins and prostatic vesicle with two portions receiving different secretions (Ogren and Kawakatsu 1990, Carbayo et al. 2013). *Pasipha ferrariaphila* also shows a folded male atrium and the female canal presenting a post-flex condition with ventral approach, i.e., the ovovitelline ducts join behind the female atrium and the female canal arises from posterior region of the female canal.

With eyes spreading over the dorsal surface of the body and a collar-shaped pharynx, *P. ferrariaphila* resembles five other species of *Pasipha*, namely *P. plana* (Schirch, 1929), *P. penhana* (Riester, 1938), *P. velutina* (Riester, 1938), *P. rosea* (E.M. Froehlich, 1955) and *P. hauseri* (Froehlich, 1959). Hence, we discuss *P. ferrariaphila* in relation to these five species in the following comparative discussion.

With respect to colour pattern, by having an almost homogeneous dorsal pattern with a thin, nearly imperceptible median stripe, *P. ferrariaphila* differs from the five species mentioned above. *Pasipha penhana* and *P. hauseri* show a quite distinct median stripe, *P. plana* and *P. rosea* a light median stripe and *P. velutina* a marbled appearance (Schirch 1929, Riester 1938, E.M. Froehlich 1955, Froehlich 1959).

Concerning internal anatomy, four of these species, namely *P. plana*, *P. penhana*, *P. velutina*, and *P. rosea*, present a not-forked prostatic vesicle with two distinct regions separated by a constriction or canal (Riester 1938, E.M. Froehlich 1955) similar to *P. ferrariaphila*. In contrast, *P. hauseri* stands apart in this group by having a forked proximal portion in its prostatic vesicle (Froehlich 1959). The new species shows a large and richly folded female atrium and, as usual in Geoplaninae, testes distributed pre-pharyngeally, differing from *P. plana*, which has a small, almost unfolded female atrium and testes almost reaching the level of the copulatory organs (E.M. Froehlich 1955). By having a horizontal female canal and an almost C-shaped common glandular ovovitelline duct, *P. ferrariaphila* differs from *P. rosea*, which shows a female canal with a C-shaped appearance and an almost horizontal common glandular ovovitelline duct (E.M. Froehlich 1955). In addition, the sperm ducts open anteriorly displaced into the proximal region of the prostatic vesicle of *P. rosea*, whereas in *P. ferrariaphila*, the openings of the sperm ducts occur into the posterior part of the proximal region of the prostatic vesicle. Froehlich (1955) describes the occurrence of a distinct circular musculature in the distal portion of the male atrium, similar to a sphincter, in both *P. plana* and *P. rosea*, which does not occur in *P. ferrariaphila*. In contrast, a strong circular musculature, resembling a sphincter, occurs in the proximal part of the female atrium of *P. ferrariaphila*.

*Pasipha ferrariaphila* shows a prostatic vesicle presenting pear-shaped proximal and distal regions of similar lengths with the distal part located above the proximal, differing from *P. penhana* and *P. velutina* (Riester 1938). In *P. penhana*, the prostatic vesicle is tubular-shaped with an elongate distal region (= *drüsiger Teil des Ductus ejaculatorius* according to Riester 1938) and a shorter proximal region (= *Ductus seminalis* according to Riester 1938). In *P. velutina*, both distal and proximal regions are tubular, giving an inverted U-shape to the prostatic vesicle (Riester 1938). In addition, besides the occurrence of a highly developed circular musculature in the proximal part of the female atrium, *P. ferrariaphila* differs from all species in this group by showing a longer and more spacious ejaculatory duct, as well as male and female atria separated by a constriction.

####### Etymology.

The specific name is a composite of the Latin noun *ferraria* (iron mine) and a suffix from the Greek adjective *phílos* (affinity), referring to its sampling site.

## Supplementary Material

XML Treatment for Pasipha ferrariaphila

## References

[B1] Amaral SV Leal-Zanchet AM (2016) Two new species of *Pasipha* Ogren & Kawakatsu (Platyhelminthes: Continenticola) from areas of deciduous forest in southern Brazil. Zootaxa 4171(3): 459–474. 10.11646/zootaxa.4171.3.3 27701211

[B2] Amaral SV Ribeiro GG Valiati VH Leal-Zanchet AM (2018) Land planarians look-alike: revealing new cryptic species on an integrative taxonomic approach. Invertebrate Systematics 32: 533–550. 10.1071/IS17046

[B3] Auler A (2015) Cavernas da Serra do Espinhaço Meridional. In: Auler A, Alt L, Moura V, Leão M (Eds) Carste Ciência e Ambiente, Minas Gerais, 17–69.

[B4] Carbayo F Álvarez-Presas M Olivares CT Marques FPL Froehlich EM Riutort M (2013) Molecular phylogeny of Geoplaninae (Platyhelminthes) challenges current classification: proposal of taxonomic actions. Zoologica Scripta 42(5): 508–528. 10.1111/zsc.12019

[B5] Froehlich CG (1959) On geoplanids from Brazil. Boletim da Faculdade de Filosofia, Ciências e Letras da Universidade de São Paulo, Ser Zoologia 22: 201–265.

[B6] Froehlich EM (1955) Sôbre espécies brasileiras do gênero *Geoplana*. Boletim da Faculdade de Filosofia, Ciências e Letras da Universidade de São Paulo, Ser. Zoologia 19: 289–369.

[B7] Froehlich EM Froehlich CG (1972) Land planarians from the Amazonian region. Papéis Avulsos do Departamento de Zoologia 26(2): 29–45.

[B8] Giulietti AM Harley RM Queiroz LP Wanderley MGL Pirani JR (2000) Caracterização e endemismos nos campos rupestres da Cadeia do Espinhaço. In: Cavalcanti TB, Walter BMT (Orgs) Tópicos atuais em botânica. 1^st^. edn. SBB/CENARGEN, Brasilia, 311–318.

[B9] Leal-Zanchet AM Rossi I Seitenfus ALR Alvarenga J (2012) Two new species of land flatworms and comments on the genus *Pasipha* Ogren & Kawakatsu, 1990 (Platyhelminthes: Continenticola). Zootaxa 3583: 1–21.

[B10] Marcus E (1951) Sobre Turbellaria Brasileiros. Boletim da Faculdade de Filosofia, Ciências e Letras da Universidade de São Paulo, Ser. Zoologia 16: 5–215. 10.11606/issn.2526-4877.bsffclzoologia.1946.125301

[B11] Negrete L Brusa F (2016) Land flatworms of the genus *Pasipha* (Platyhelminthes, Geoplanidae) in Argentina, with description of three new species. Zootaxa 4137(2): 187–210. 10.11646/zootaxa.4137.2.2 27470715

[B12] Negrete L Brusa F (2017) Increasing diversity of land planarians (Platyhelminthes: Geoplanidae) in the Interior Atlantic Forest with the description of two new species and new records from Argentina. Zootaxa 4362(1): 99–127. 10.11646/zootaxa.4362.1.5 29245445

[B13] Ogren RE Kawakatsu M (1990) Index to the species of the family Geoplanidae (Turbellaria, Tricladida, Terricola). Part I: Geoplaninae. Bulletin of the Fuji Women’s College, Ser. 2 28: 79–166.

[B14] Oliveira PA Pereira IM Messias MCTB Oliveira MLR Pinheiro AC Machado ELM Oliveira JLA (2018) Phytosociology of the herbaceous-subshrub layer of a rupestrian complex in Serra do Espinhaço, Brazil. Acta Botanica Brasilica 32(1): 141–149. 10.1590/0102-33062017abb0225

[B15] Rapini AA Ribeiro PL Lamberti S Pirani JR (2008) A flora dos campos rupestres quartzíticos da Cadeia do Espinhaço. Megadiversidade 4: 16–24.

[B16] Riester A (1938) Beiträge zur Geoplaniden-Fauna Brasiliens. Abhandlungen der Senckenbergischen Naturforschenden Gesellschaft 441: 1–88.

[B17] Rossi I Amaral SV Ribeiro GG Cauduro GP Fick I Valiati VH Leal-Zanchet AM (2015) Two new Geoplaninae species (Platyhelminthes: Continenticola) from Southern Brazil based on an integrative taxonomic approach. Journal of Natural History 50: 1–29. 10.1080/00222933.2015.1084057

[B18] Schirch PF (1929) Sobre as planárias terrestres do Brasil. Boletim do Museu Nacional 5(1): 27–38.

